# 

**Published:** 1895-06-22

**Authors:** 


### CONTENTS.

The Physiology and Cure of Stuttering . . . . 195

The Certificate Nuisance . . . . . . . . 196

On Modern Progress in Ophthalmic Medicine and Surgery. By ROBERT BRUDENELL CARTER, F.R.C.S. . . 197

The Medical Profession. By Ven. W. M. SINCLAIR, D.D., Archdeacon of London . . . . . . . 198

Annotations.—Doctor or Schoolmaster?—The W. G. Grace Tribute Frozen Milk . . . . . . . . . . 199

Notes and News . . . . . . . . . . . . 200

Medical Progress and Hospital Clinics—

The Treatment of Sprains and Fractures in the Neighbourhood of Joints by Massage and Early Passive Movements . . 201

PROGRESS IN MEDICINE.—Diseases of Children . . . . 203

NEW APPLIANCES AND THINGS MEDICAL . . . . . 204

The Institutional Workshop—

HOSPITAL SUNDAY IN LONDON . . . . . . . 205

DISTRIBUTING THE SPECIAL SUPPLEMENT . . . . . 207

HOSPITAL FESTIVALS AND MEETINGS . . . . . . 209

### EXTRA SUPPLEMENT.—THE NURSING MIRROR.

News from the Nursing World.—District Nursing at Kensington — University College Hospital Nurses — Help Those Who Help Themselves—Training at Tottenham—An Excellent Example—Nursing in Cottage Hospitals—The Troubles at Macclesfield—A Fête at Cold Ash—A Satisfactory Arrangement—Queen's Nurses at Darlington—The Work of a Woman—Pensions for American Nurses—Short Items . lxxvi

Elementary Anatomy and Surgery for Nurses. By W. MCADAM ECCLES, M.D., M.S., F.R.C.S. XXII.—Surgical Affections of Respiratory Passages . . . . . . lxxix

Appointments . . . . . . . . . . lxxix

Minor Appointments . . . . . . . . . lxxix

National Society for the Prevention of Cruelty to Children . . . . . . . . . . lxxx

Medico-Psychological Association . . . . . . lxxx

Death in Our Ranks . . . . . . . . lxxx

Hospital Sunday . . . . . . . . . lxxx

Royal British Nurses' Association . . . . . . lxxx

Everybody's Opinion . . . . . . . . lxxxi

Where to Go . . . . . . . . . . lxxxi

Notes and Queries . . . . . . . . . lxxxi

Wants and Workers . . . . . . . . lxxxi

Novelties for Nurses . . . . . . . . lxxxii

The Book World for Women and Nurses . . . . lxxxii

